# NLLSS: Predicting Synergistic Drug Combinations Based on Semi-supervised Learning

**DOI:** 10.1371/journal.pcbi.1004975

**Published:** 2016-07-14

**Authors:** Xing Chen, Biao Ren, Ming Chen, Quanxin Wang, Lixin Zhang, Guiying Yan

**Affiliations:** 1 School of Information and Electrical Engineering, China University of Mining and Technology, Xuzhou, China; 2 Chinese Academy of Sciences Key Laboratory of Pathogenic Microbiology and Immunology, Institute of Microbiology, Chinese Academy of Sciences, Beijing, China; 3 State Key Laboratory of Oral Diseases, West China Hospital of Stomatology, Sichuan University, Sichuan, China; 4 University of Chinese Academy of Sciences, Beijing, China; 5 South China Sea Institute of Oceanology, Chinese Academy of Sciences, Guangzhou, China; 6 Academy of Mathematics and Systems Science, Chinese Academy of Sciences, Beijing, China; University of Calgary Cumming School of Medicine, CANADA

## Abstract

Fungal infection has become one of the leading causes of hospital-acquired infections with high mortality rates. Furthermore, drug resistance is common for fungus-causing diseases. Synergistic drug combinations could provide an effective strategy to overcome drug resistance. Meanwhile, synergistic drug combinations can increase treatment efficacy and decrease drug dosage to avoid toxicity. Therefore, computational prediction of synergistic drug combinations for fungus-causing diseases becomes attractive. In this study, we proposed similar nature of drug combinations: principal drugs which obtain synergistic effect with similar adjuvant drugs are often similar and vice versa. Furthermore, we developed a novel algorithm termed Network-based Laplacian regularized Least Square Synergistic drug combination prediction (NLLSS) to predict potential synergistic drug combinations by integrating different kinds of information such as known synergistic drug combinations, drug-target interactions, and drug chemical structures. We applied NLLSS to predict antifungal synergistic drug combinations and showed that it achieved excellent performance both in terms of cross validation and independent prediction. Finally, we performed biological experiments for fungal pathogen Candida albicans to confirm 7 out of 13 predicted antifungal synergistic drug combinations. NLLSS provides an efficient strategy to identify potential synergistic antifungal combinations.

## Author Summary

Drug combinations represent a promising strategy for overcoming fungal drug resistance and treating complex diseases. There is an urgent need to establish powerful computational methods for systematic prediction of synergistic drug combination on a large scale. Based on the assumption that principal drugs which obtain synergistic effect with similar adjuvant drugs are often similar and vice versa, NLLSS was developed to predict potential synergistic drug combinations by integrating known synergistic drug combinations, unlabeled drug combinations, drug-target interactions, and drug chemical structures. NLLSS has obtained the reliable performance in the cross validation and experimental validations, which indicated that NLLSS has an excellent performance of identifying potential synergistic drug combinations. Out of 13 predicted antifungal synergistic drug combinations, 7 candidates were experimentally confirmed. It is anticipated that NLLSS would be an important and useful resource by providing a new strategy to identify potential synergistic antifungal combinations, explore new indications of existing drugs, and provide useful insights into the underlying molecular mechanisms of synergistic drug combinations.

## Introduction

In recent years, fungal infection has become one of the leading causes of hospital-acquired infections with high mortality rates due to growing populations of patients with weakened immune systems, for example due to cancer, organ transplant or Acquired Immune Deficiency Syndrome (AIDS). In these patients, infections caused by *Candida*, *Aspergillus* and *Cryptococcus neoformans* fungi strains may take the form of potentially lethal blood stream infections, lung infections and other infections. For example, *Candida* causes candidiasis, which becomes the fourth most common fungal blood stream infection among hospitalized patients in the United States according to the Centers for Disease Control & Prevention. Unfortunately, fungal infections that include *Candida albicans* have become resistant to current drug treatments. Therefore, there is an urgent need to develop new therapies to overcome the drug resistance and kill *C*. *albicans*.

Drug combinations have been widely used to overcome drug resistance and treat complex disease such as cancer and infectious diseases [1–4]. Drug combinational treatment could inhibit new multiple targets and thus provide the opportunity for overcoming drug resistances of infectious fungi [5–7]. The potential molecular mechanism underlying this is that biological systems are less able to compensate for the simultaneous activity of two or more drugs [1,5,8,9]. Indeed, we have seen growing enthusiasm over the development of synergistic drug combinations in academia, as well as the pharmaceutical industry. For example, CRx-102 is a novel synergistic drug candidate combination comprised of dipyridamole and low-dose prednisolone. This drug combination can be used for the treatment of osteoarthritis (OA) and has already completed Phase II study in Knee OA [10]. Also, moduretic, a combination of Amiloride and Hydrochlorothiazide, is used to treat patients with hypertension [11,12]. The use of synergistic drug combinations can increase treatment efficacy and decrease drug dosage to avoid toxicity. It also has been pointed out that off-target effects could be overcome by drug combinations [13]. These advantages have increasingly driven researchers towards the search for safe and effective combinatorial drugs [5–7,14].

Traditionally, effective drug combinations have been identified through experimentally screening all possible combinations of a pre-defined set of drugs [5,15]. Given the large number of drugs, experimental screens of pairwise combinations of drugs will be cost expensive, time consuming and labor intensive. For example, given *n* drugs, there will be *n*(*n*−1)/2 pairwise drug combinations and many more higher-order combinations. Furthermore, new drugs will be produced every year, therefore, the number of possible drug combinations will exponentially increase [15]. Since a comparatively small number of compounds will provide a very large number of combinations [6], experimentally testing all the possible drug combinations would pose a formidable challenge in terms of cost and time. Even when high-throughput screens are adopted, limited drug combination experiments would only sample a small fraction of so many candidate drug combinations. Thus, it is not easy to identify optimal drug combinations using the experimental screen approach [16]. To overcome this problem, we intend to develop a method that computationally ‘screens’ synergistic drug combinations and identifies optimal drug pairs for treating drug resistance of fungus-infected diseases. Our computational methods can select the most promising drug combinations for rigorous validation through biological experimentation, thus saving time and money. In this sense, this method could guide the drug combinations experiments and also benefit the understanding of mechanisms underlying synergistic drug combinations.

Previous research was mostly focused on defining the concept of synergy, quantitatively measuring dose-effect curves, and determining whether or not a given drug combination could achieve synergistic effect according to the definitions of the synergy and experiment results [1]. Ever since Loewe proposed the Loewe additive model to describe synergy drug combination in 1928, numerous researchers have devoted to drug combination analysis [1,14,17–22]. Loewe defined Loewe additive equation as follows to determine whether or not the given drug combination would result in a synergistic effect [17,18]:

$$\frac{{\left(D\right)}_{1}}{{\left(D_{x}\right)}_{1}}+\frac{{\left(D\right)}_{2}}{{\left(D_{x}\right)}_{2}}=1$$

Variables in the numerator are the dosage of each drug (drug ‘1’ and drug ‘2’) when these two drugs are combined and x% is the inhibition rate with this concentration combination. Variables in the denominator are the dosage of each drug that can inhibit the system by x%. The left-hand side of this equation is less than 1 and more than 1 mean Loewe synergism and Loewe antagonism, respectively. Then, Bliss defined the expected combination effect as *I*_*Mult*_ = *I*_*X*_ + *I*_*Y*_ − *I*_*X*_*I*_*Y*_, where *I*_*X*_ and *I*_*Y*_ are single drug inhibition at concentrations X and Y [19]. Berenbaum proposed the highest single agent (HSA) model, which defined the expected response as *I*_*HSA*_ = max{*I*_*X*_, *I*_*Y*_}, where *I*_*X*_ and *I*_*Y*_ are defined in a manner similar to that of the Bliss model [23]. Chou and Talalay proposed the median-effect equation [21,24,25], the Combination Index (CI)-Isobologram equation [20,21], and the dose-reduction index equation [21,26] for quantitative determination of drug combination interactions. In their scheme, *CI*<1, = 1, and >1 indicate synergism, additive effect, and antagonism, respectively [1]. Greco also established a new method, termed universal response surface approach (URSA), for the quantitative assessment of drug interactions [27]. However, all aforementioned models only determine whether or not a given drug combination could achieve synergistic effect and can’t be used to predict potential synergistic drug combinations.

In recent years, some methods have been developed to decrease the number of drug combination experiments. Jansen et al. [14] used chemogenomic profiles to identify potential combinatorial drugs. Firstly, sensitivity-based chemogenomic profile data generated from the literature and profiling experiments were analyzed. Then, any given compound pair that had chemogenomic profiles similar to the known synergy pairs was considered as potential antifungal synergy candidates. Chen et al. [28] combined fractional factorial design and stepwise regression to dramatically reduce the time of experiments required to identify synergistic drug combinations. However, these two methods both strongly rely on biological experimental results. Li et al. [29] defined the parameters of topology score and agent score to evaluate the synergistic relationship for given drug combinations and further established the algorithm termed NIMS to uncover potential synergistic drug combinations on a large scale. Zhao et al. [30] represented drugs based on a set of their properties and further developed a novel computational method to prioritize candidate drug combinations by integrating molecular and pharmacological data. Huang et al. [31] integrated clinical side-effect information and the drug label to predict drug combination and demonstrated that three FDA black-box warned serious side-effects contributed mostly to the prediction performance. Huang et al. [32] developed a computational synergistic drug combination prioritization tool (DrugComboRanker) based on drug functional network construction and partition. Yin et al. [33] shown drug synergy or antagonism to be a property of target-related network topology and analyzed several basic synergistic and antagonistic motifs to indicate that designing novel synergistic drug combinations based on network topology could be promising. Iwata et al [34] integrated known synergistic drug combinations from the Orange Book and KEGG DRUG database, drug-target interactions, and drug Anatomical Therapeutic Chemical Classification System codes to construct a sparsity-induced classifier for the potential synergistic drug combination inference. Recently, considering the important fact that synergistic drug combination may act on the same pathway through different drug targets, Chen et al. [35] included the information of systematic pathway-pathway interactions and further developed a novel network-based synergistic drug combination prediction model. However, only computational models have been developed and no experimental validation could be found in aforementioned seven studies.

Thus, in this study, we developed a novel algorithm, called Network-based Laplacian regularized Least Square Synergistic drug combination prediction method (NLLSS), to conduct computational ‘screens’ by integrating several types of information such as known synergistic drug combinations, unlabeled combinations (all the drug combinations without known synergistic evidences), drug-target interactions, and drug chemical structures. NLLSS obtained excellent performance in both cross validation and independent antifungal drug combinations prediction. Furthermore, we experimentally confirmed 7 out of 13 predicted antifungal synergistic drug combinations for fungal pathogen *Candida albicans*. These combinations could provide new treatments for overcoming fungal drug resistance. Finally, NLLSS provides an efficient strategy to find potential synergistic antifungal combinations by exploring new indications of existing antifungal drugs. Further, NLLSS could also be used for predicting synergistic drug combinations for treating other diseases.

## Materials and Methods

### Antifungal synergistic drug combinations

First, we investigated hundreds of studies on drug combinations and selected 69 compounds involved in antifungal drug combination experiments (see S1 Table). Then we searched literatures with the keywords ‘synergy’, ‘synergic’, ‘synergistic’, ‘synergism’, ‘interaction’ and ‘combination’ in the PubMed, Google Scholar and Web of Knowledge and collected 75 experimentally confirmed synergistic antifungal drug combinations (dataset 1, see S2 Table). Therefore, all the antifungal compounds involved in antifungal drug combination experiments are considered in this study. We do not require they must have known synergistic partners. We also classified these compounds into principal drugs and adjuvant drugs according to the following rules. If one compound in the synergistic combination shows activity in the antifungal assay, but the other does not, as reported before, then the former compound is considered as the principal drug, and the latter is considered as the adjuvant drug. If both compounds in the synergistic combination show activity in the antifungal assay, or neither one shows activity, as reported before, then these two compounds are considered as both principal and adjuvant drug. If one compound does not have antifungal synergistic effect with any other compound, then this compound is classified according to its antifungal activity.

To further confirm the predictive ability of NLLSS, we further obtained two other antifungal drug combination datasets from the dataset 1: synergistic antifungal drug combinations against *Candida albicans* (dataset 2, the drugs are the same as the ones in dataset 1 and the combinations are the ones in dataset 1 against *Candida albicans*) and synergistic azole drug combinations (dataset 3, the drugs are the ones in dataset 1 which are azole drug and the combinations are the azole drug combinations in dataset 1). Related information on these two synergistic drug combination datasets can be obtained from the supplementary materials (See S3–S6 Tables: drugs and synergistic drug combinations in dataset 2 and 3, respectively). Statistics of three drug combination datasets were listed in Table 1, including the number of drugs, principal drugs, adjuvant drugs, known synergistic combinations (A), and drug pairs without known synergistic relationship (B) and the ratio A/B.

**Table 1:** Statistics of three drug combination datasets were listed, including the number of drugs, principal drugs, adjuvant drugs, known synergistic combinations (A), and drug pairs without known synergistic relationship (B) and the ratio A/B.

	Drug	Principal drug	Adjuvant drug	Known synergistic combination (A)	Drug pairs without known synergistic relationship (B)	A/B
1	69	62	67	75	4079	0.0184
2	69	62	67	71	4083	0.0174
3	55	49	52	73	2475	0.0295

In this study, NLLSS was developed based on the framework of Laplacian Regularized Least Square (LapRLS), which required drugs in the same combination must be divided into principal drug and adjuvant drug. However, according to the classification rules mentioned before, many drugs have been considered as both principal and adjuvant drugs. This fact ensure that we still can obtain plenty of potential synergistic drug combinations composed of two principal drugs or two adjuvant drugs.

### Drug chemical structure similarity

Chemical structure similarities between compounds were calculated by SIMCOMP [36] based on chemical structure information from the DRUG and COMPOUND Sections in the KEGG LIGAND database [37]. The similarity calculated from SIMCOMP is a global score based on the ratio between the size of the common substructures and the size of the union structures [36]. Applying this operation to all compound pairs, chemical structure similarity scores between principal (adjuvant) drugs can be obtained

### Drug-target interactions

Target proteins of all the drugs in three drug combination datasets were obtained from the Drug Bank database [38] and related literatures. The drug-protein interactions in three datasets were shown in S7–S9 Tables, respectively.

### Drugs for biological experiments

Ketoconazole, Fluconazole, Voriconazole, Posaconazole, Itraconazole, Terbinafine, Flucytosine, Radicicol, Disulfiram, Lovastatin, Geldanamycin, Caspofungin and Micafungin were purchased from a local pharmaceutical company. Amphotericin B, Beauvericin and FK506 were purchased from Sigma.

### Principles of NLLSS

**Fig 1:**
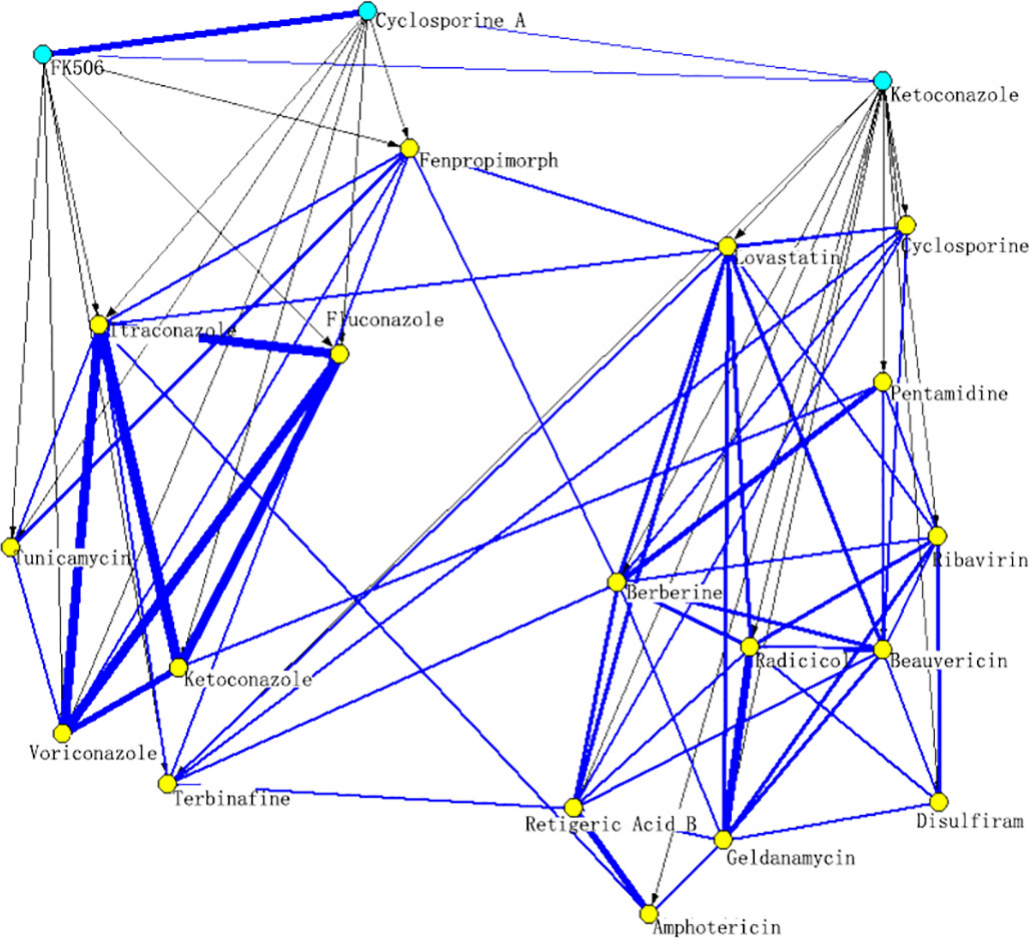
Schematic illustrating the similar nature of synergistic drug combinations. Blue nodes represent principal drugs, and yellow nodes represent adjuvant drugs. The arc from the principal drug to the adjuvant drug means that two drugs have synergistic effect when combined in the antifungal assays. The edge between two principal (adjuvant) drugs represents the similarity between two drugs. Thickness of edges linking drugs indicates degree of similarity between them. This figure shows adjuvant drugs which obtain synergistic effect with similar principal drugs are often similar. Similar principal drugs, Cyclosporine A and FK 506 gain synergistic effect with seven adjuvant drugs, including Fenpropimorph, Fluconazole, Itraconazole, Ketoconazole, Tunicamycin, Voriconazole, and Terbinafine. It can be observed that those seven adjuvant drugs are similar. Also the adjuvant drugs which obtain synergistic effect with Ketoconazole are similar and form a module. On the contrary, most of the adjuvant drugs which obtain synergistic effect with dissimilar principal drugs (Cyclosporine A and Ketoconazole, FK506 and Ketoconazole) are dissimilar.

**Fig 2:**
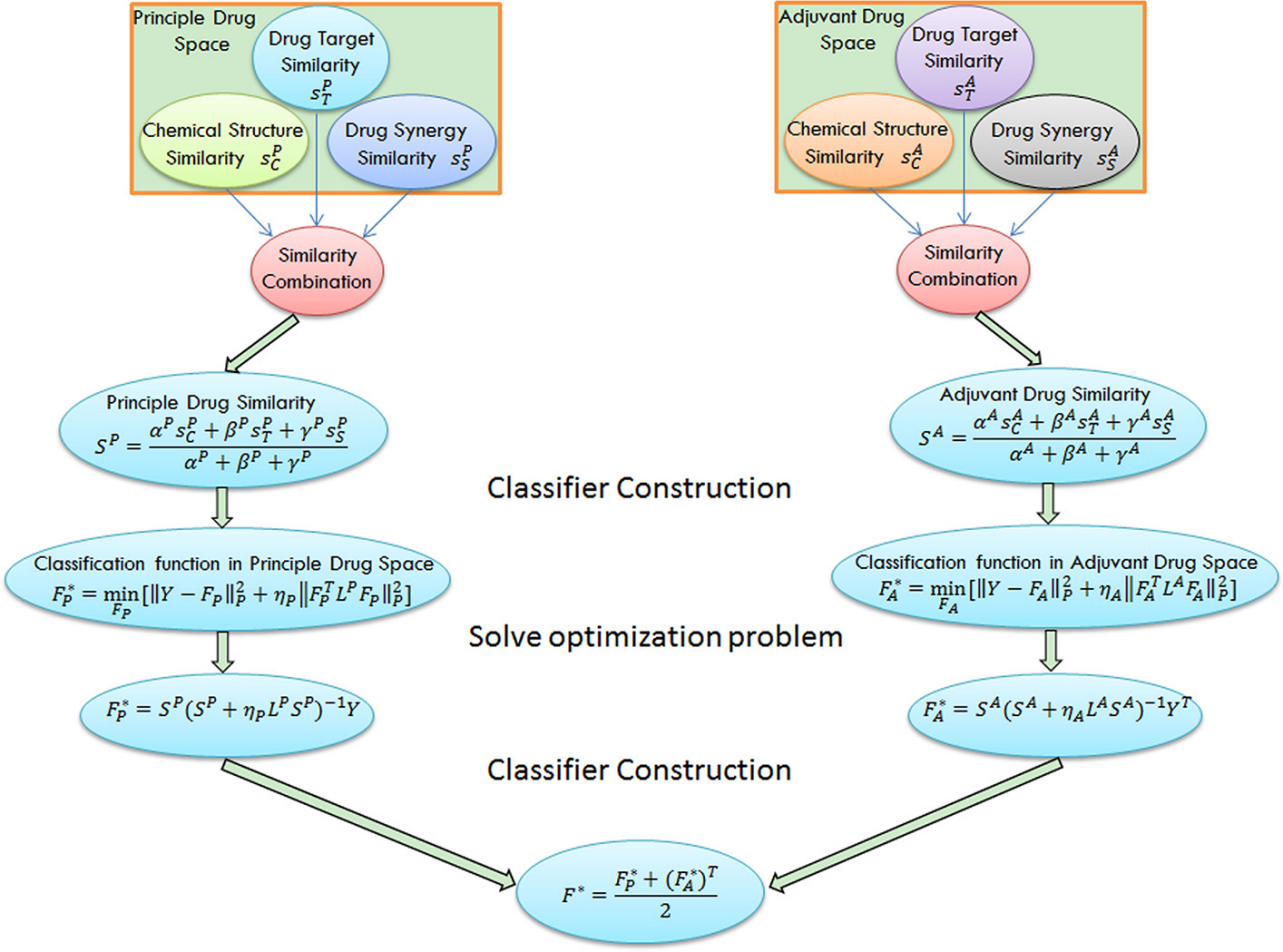
The basic idea of NLLSS is shown. First, the principal and adjuvant drug similarity are calculated based on drug chemical structure, drug-target interactions, and known synergistic drug combinations. Next, we construct the synergistic drug combination classifiers in the principal and adjuvant spaces, respectively. Finally, two classifiers are combined into the final classifier to select potential synergistic drug combinations for experimental validation.

NLLSS assumes that principal drugs which obtain synergistic effect with similar adjuvant drugs are often similar and vice versa, which is referred to as the similar nature of drug combinations (see the examples in Fig 1 and S1 Fig). Based on this assumption, the following conclusion can be deduced: principal drugs which obtain synergistic effect with the same adjuvant drug are often similar and vice versa. In this paper, similarity between two drugs is established on the basis of drug chemical structure, drug-target interactions, and known synergistic drug combinations. The similar nature of drug combination was further formulated into two classifiers based on the framework of LapRLS in the principal and adjuvant drug space, respectively. The classifiers in the principal drug space and adjuvant drug space both considered all possible drug pairs. They both used the information of known synergistic drug combinations and unlabeled drug combinations. The difference between these two classifiers was that they adopted the different drug similarity. Classifier in the principal drug space only used principal drug similarity. Correspondingly, classifier in the adjuvant drug space only used the information of adjuvant drug similarity. Finally, two classifiers were combined into a single classifier to give a final predictive result. Based on the model, a score to assess how likely two drugs will obtain synergistic effect can be obtained. Drug combination pairs with high scores can be expected to have a high probability of obtaining synergistic effect when combined, thus having priority in subsequent biological experiments and, in turn, reducing the cost of identifying potential synergistic drug combinations. The flow chart of NLLSS is shown in Fig 2.

### Drug similarity calculation

NLLSS first calculates the similarity between drugs. In this model, the similarity between two drugs depends on three factors: drug chemical structure similarity, drug target similarity, and drug synergistic similarity.

**Fig 3:**
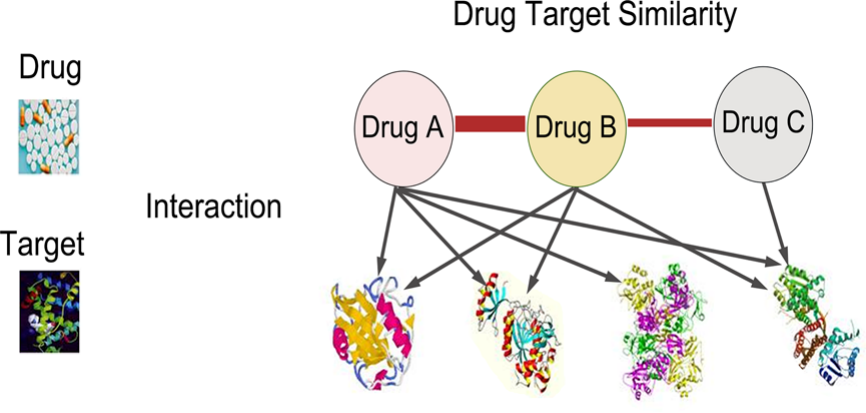
In order to calculate the similarity between two drugs, we extract the information from drug-target interactions. The underlying assumption made here is that two drugs that share more common target proteins are more similar.

Drug chemical structure similarity can be obtained by SIMCOMP as noted before. We defined principal (adjuvant) drug chemical structure similarity matrix as $S_{C}^{P}(S_{C}^{A})$. Next we want to extract the information from drug-target interactions for the measurement of drug similarity. The underlying assumption made here was that two drugs are similar if they share more common target proteins (see Fig 3). Based on this assumption, the principal (adjuvant) drug target similarity matrix $S_{T}^{P}(S_{T}^{A})$ was defined. The entity of the matrix was the number of target proteins shared by two drugs. Third, we extracted the information from known drug synergistic combinations, assuming that if two principal (adjuvant) drugs obtain synergistic effect with more common adjuvant (principal) drugs, they will have greater similarity (see S2 Fig). The principal (adjuvant) drug synergistic similarity matrix was defined as $S_{S}^{P}(S_{S}^{A})$. The entity of the matrix was the number of common adjuvant (principal) drugs which have synergistic effect with two principal (adjuvant) drugs.

Drug-target similarity matrix and drug synergistic similarity matrix must be normalized. For $S_{T}^{P}$, we defined a diagonal matrix $D_{T}^{P}$ such that $D_{T}^{P}(i,i)$ was the sum of row *i* of $S_{T}^{P}$. We set STP'=(DTP)−1/2STP(DTP)−1/2 which yielded a symmetric matrix where STP'(i,j)=STP(i,j)/DTP(i,i)DTP(j,j). A similar operation was applied to another three matrices. Now, the principal drug similarity matrix *S*^*P*^ can be obtained by linear combination as follows:

SP=αPSCP+βPSTP'+γPSSP'αP+βP+γP

where combinatorial coefficient means the weight of various similarity measures for the final integrated principal drug similarity. Similarly, the adjuvant drug similarity matrix *S*^*A*^ can be obtained by the following form.

SA=αASCA+βASTA'+γASSA'αA+βA+γA

Here, we have adopted the method of weighted averaging for the drug similarity integration, which means all the drug similar measures have equal weight (i.e. *α*^*P*^ = *β*^*P*^ = *γ*^*P*^ = 1/3, *α*^*A*^ = *β*^*A*^ = *γ*^*A*^ = 1/3) for the final drug similarity matrix.

### Construction of the classifier

For the employment of the LapRLS, Laplacian operation must be applied to the similarity matrix. The diagonal matrices *D*^*P*^ and *D*^*A*^ were defined such that *D*^*P*^(*i*,*i*) and *D*^*A*^(*i*,*i*) were the sum of row *i* of *S*^*P*^ and *S*^*A*^*S*^*A*^, respectively. The normalized Laplacian matrices were defined as follows:

$$\begin{array}{l} L^{P}={\left(D^{P}\right)}^{-1/2}(D^{P}-S^{P}){\left(D^{P}\right)}^{-1/2}\\ L^{A}={\left(D^{A}\right)}^{-1/2}(D^{A}-S^{A}){\left(D^{A}\right)}^{-1/2} \end{array}$$

Let matrix *Y* represents prior synergistic drug combination information. If principal drug *i* and adjuvant drug *j* were known to produce synergistic effect, then *Y*(*i*,*j*) = 1; otherwise *Y*(*i*,*j*) = 0. The aim was to obtain a continuous classification function, which reflected the probability that two drugs could obtain synergistic effect when combined. Intuitively, it is anticipated that when similar principal (adjuvant) drugs are combined with the same adjuvant (principal) drug, these combinations can obtain similar synergistic probability scores. Also this classification function should comply with prior synergistic information. LapRLS defines a cost function and wants to minimize this cost function in order to obtain an optimal classification function. The classification function was composed of optimal functions in the principal drug space and adjuvant drug space.

We first address how to obtain optimal classification function in the principal drug space. Cost function was defined as follows:

$$F_{p}^{*}=\underset{F_{P}}{\text{arg min}}[{\left\|Y-F_{P}\right\|}_{F}^{2}+\eta_{P}{\left\|F_{p}^{T}L^{P}F_{P}\right\|}_{F}^{2}]$$

Where ‖.‖_*F*_ is Frobenius norm and *η*_*P*_ is the trade-off parameter in the principal drug space. Then, we can get the optimal classification function [39,40] as follows:

$$F_{P}^{*}=S^{P}{\left(S^{P}+\eta_{P}L^{P}S^{P}\right)}^{-1}Y$$

We also can get the optimal classification function in adjuvant drug space in a similar manner:

$$F_{A}^{*}=S^{A}{\left(S^{A}+\eta_{A}L^{A}S^{A}\right)}^{-1}Y^{T}$$

where *η*_*A*_ is the trade-off parameter in the adjuvant drug space. We set these two trade-off parameters as 0.3 in this study according to previous literatures [40–43]. Hence, the classification function can be obtained by combining the prediction results in both principal and adjuvant drug space, as follows:

$$F^{*}=\frac{F_{P}^{*}+{\left(F_{A}^{*}\right)}^{T}}{2}$$

We converted the probability of candidate drug combinations to the Rank Probability (RP). The probabilities of combinations were ranked in ascending order, and each candidate combination obtained Rank (R). Rank Probability (RP) of a drug combination was calculated by Rank (R) divided by the total number of candidate drug combinations (N). In this case, the most probable synergistic drug combination will get the RP of 1.

### Synergistic antifungal bioassay

*Candida albicans* SC5314 was used as a test strain for antifungal and synergistic antifungal bioassay. All procedures were described previously [16]. The experiments were carried out in flat bottom, 96-well microtiter plates (Greiner), using a broth microdilution protocol modified from Clinical and Laboratory Standards Institute M-27A methods [44]. Overnight cultures were selected to prepare the strain suspension with RPMI 1640 medium (Gibco) at the concentration of 1×10^4^ cells/mL counted by hemocytometer. To the test wells in 96-well plates, 2 μL of the samples were added, followed by an additional 80 μL of the strain suspension. The test plates were incubated at 35°C. The antifungal MICs were determined by measuring and comparing the optical densities of the positive control and test wells at different time points. For the synergistic antifungal assay, checkerboard assay was used, and beauvericin combined with ketoconazole served as positive control [16]. The MICs were determined by measuring and comparing the optical densities of the positive control and test wells at different time points.

## Results

### Cross validation

**Fig 4:**
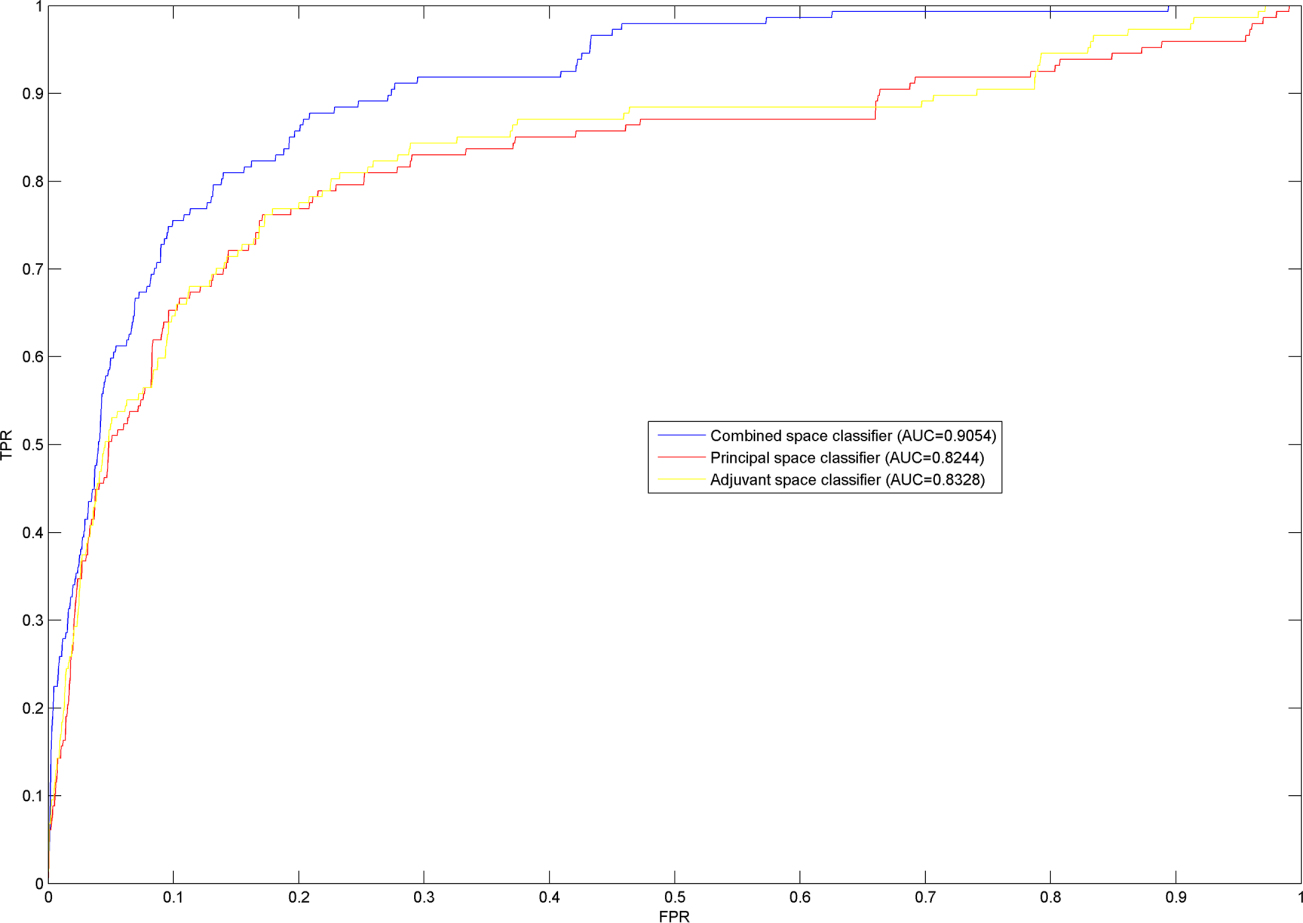
LOOCV was implemented in the antifungal synergistic drug combinations dataset 1. AUC was calculated to evaluate the performance of the method. Here known synergistic drug combinations were used as a test dataset. The ROC curves of NLLSS based on the combination of two classifiers and based only on a single classifier were compared. The results confirmed the performance advantage of combining the classifiers in the principal and adjuvant drug space into a single classifier.

**Fig 5:**
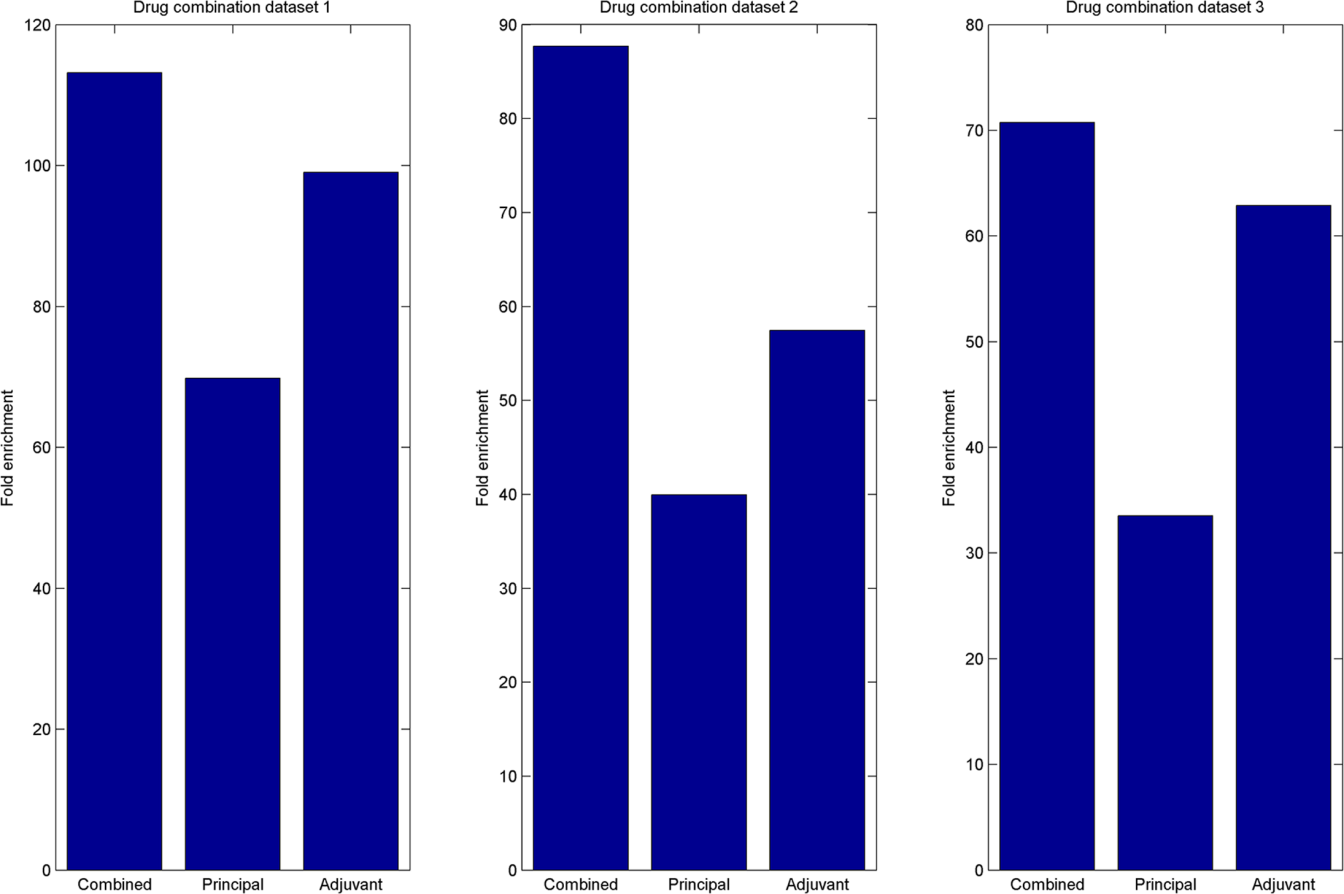
LOOCV was implemented in three antifungal synergistic drug combinations datasets. Fold enrichment score was calculated to evaluate the performance of the method. Here known synergistic drug combinations were used as a test dataset. The fold enrichment score of NLLSS based on the combination of two classifiers and based only on a single classifier were compared. The results confirmed the performance advantage of combining the classifiers in the principal and adjuvant drug space into a single classifier.

We evaluated the predictive performance of NLLSS using leave-one-out cross validation (LOOCV). To do so, each known synergistic drug combination was treated as a test dataset in turn, while the remaining known synergistic drug combinations were used as the training dataset. First, we calculated the enrichment score to measure the performance of NLLSS. When LOOCV is implemented, if there are *n* candidate drug combinations without known synergistic evidences, the enrichment score is calculated by dividing n/2 by the rank of the left-out drug combination among candidate drug combinations. For example, if NLLSS gives the left-out known synergistic drug combination the highest ranking (ranked 1st in the candidate drug combinations), there would be an enrichment score of n/2. Furthermore, if the left-out known synergistic drug combination is ranked by random, it would have the rank of n/2 and therefore have an enrichment score of 1. Therefore, enrichment score could represent the difference between prediction accuracy obtained by NLLSS and random. Here, the average of enrichment scores for all the left-out known combinations is calculated for the final evaluation. Next, receiver-operating characteristic (ROC) curve was used as another evaluative measure. The ROC curve plots the true-positive rate (TPR) versus the false-positive rate (FPR). The area under the ROC curve (AUC) was calculated to reflect predictive accuracy. Here, the ROC curves of NLLSS based on the combination of two classifiers and only based on a single classifier in three drug combination datasets were compared (Fig 4, S3 Fig, S4 Fig). The AUCs for the combination of two classifiers in three dataset were 0.9054, 0.8963, and 0.8819, respectively, which shows reliable ability to predict potential synergistic drug combinations. The AUCs for the classifier in the principal and adjuvant drug space were significantly inferior to the combined classifier, which shows the reasonableness of combining the classifiers in the principal and adjuvant drug space. Also, the comparisons of combined and single classifiers in terms of fold enrichment score in the three drug combination datasets were shown in Fig 5, still illustrating the prefect performance of NLLSS.

Performance comparison between NLLSS with current state-of-the-art computational models can’t be reasonably implemented. Different synergistic drug combination benchmark datasets and drug data sources for drug similarity calculation have been used in the different studies. For example, we only paid attention to antifungal synergistic drug combinations and integrated drug chemical structure, drug-target interactions, and known synergistic drug combinations to calculate drug similarity. In order to compare different computational models based on the same benchmark dataset, we must obtain different data sources of all the drugs in the benchmark dataset, such as drug-target interactions, drug side-effect information, and drug chemical structure. It is difficult to obtain all the datasets. Furthermore, some studies didn’t use known synergistic drug combinations to predict potential ones. It is unreasonable to directly compare them with NLLSS. The AUCs of NLLSS was 0.9054, which has been better than AUCs reported in the previous studies.

Furthermore, in order to confirm NLLSS is robust to the training sample selection, we implemented 10-fold, 5-fold, and 3-fold cross validation (CV) in all the three datasets, respectively (See Table 2). Here, all the known synergistic combinations were randomly divided into 10-fold, 5-fold, and 3-fold, which means 90%, 80%, and 66.67% of the known synergistic combinations were regarded as the training samples for model learning and the other 10%, 20%, and 33.3% were used as test samples for performance validation, respectively. Considering the potential influence caused by sample division, we implemented 100 different random divisions and calculated the mean and the standard deviation of all the obtained AUCs. As the results listed in Table 2, NLLSS has a reliable and robust performance in all the validation schemas.

**Table 2:** Performance of NLLSS under the validation framework of LOOCV, 10-fold CV, 5-fold CV, and 3-fold CV, which have demonstrated the NLLSS has a reliable and robust performance.

Dataset	LOOCV	10-fold CV	5-fold CV	3-fold CV
Dataset 1	0.9054	0.8965+/-0.0066	0.8832+/-0.0095	0.8692+/-0.0119
Dataset 2	0.8963	0.8832+/-0.0077	0.8750+/-0.0110	0.8545+/-0.0131
Dataset 3	0.8819	0.8718+/-0.0066	0.8574+/-0.0112	0.8380+/-0.0139

#### Predict potential synergistic antifungal drug combinations using NLLSS

NLLSS was applied to identify potential antifungal synergistic drug combinations. Known synergistic antifungal combinations in datasets 1, 2, and 3 were used as the training set, respectively. Complete predictive results for three datasets were listed in S10–S12 Tables, respectively.

### Experimental validation of the antifungal drug combinations in fungal pathogen *Candida albicans*

**Fig 6:**
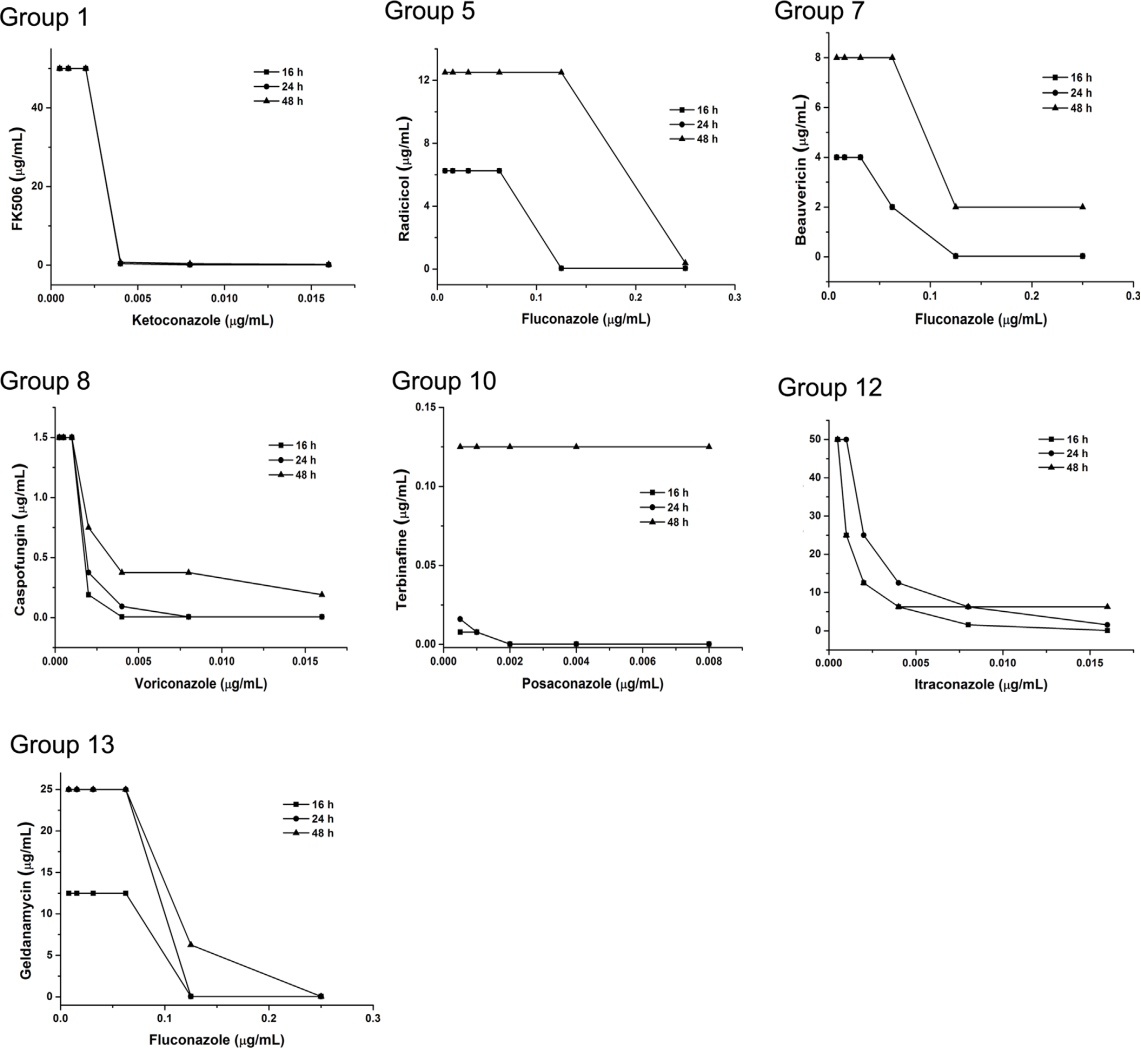
Synergistic antifungal combinations: The x and y axis indicated the concentrations of the combination drugs used in the synergistic screening. The dots were the active concentrations for inhibiting the growth of C. albicans in the combinations. All experiments were performed on 96-well plates and incubated at 35 oC for 48 h. The data from three independent experiments were measured at different time points (16 h, 24 h and 48 h).

To experimentally validate the predicted combinations which have potential synergistic antifungal activities, we tested all combinations *in vitro* on the leading human pathogen *Candida albicans*. Here, we implemented experiments for the top 10 potential drug combinations in all the three datasets (See Table 3). The synergis tic activities were judged by fractional inhibitory concentration index (FICI) values, which were calculated by comparing MICs in combinations with MICs of the each drug used alone (See S13 Table) at different time points. Biological experimental results indicated that 6, 5, and 6 out of the top 10 potential combinations in three datasets did indeed obtain antifungal synergistic effect (See Table 3). After the removal of duplicate combinations, we found 7 synergistic combinations (Fig 6) and proved that 6 groups were nonsynergistic combinations (S5 Fig) in total. Considering the validated 7 synergistic combinations are totally new combinations, which have not been reported in any publicly published literatures, this prediction accuracy could be considered high. To identify 7 synergistic combinations, we only need implement experiments for 13 candidate combinations, which have greatly reduced the cost and time of pure experimental research. Most of previous computational studies for synergistic drug combination prediction didn’t implement any experimental validation. Only Jansen et al (2009) implemented biological experiments for predicted synergistic combination. However, training samples and criterion of selecting potential combinations for experimental validation were totally different between their studies and NLLSS. Therefore, it is also difficult to compare these two models based on the accuracy of independent prediction.

**Table 3:** Biological experimental results for potential top 10 antifungal drug combinations in three drug combination datasets, and the targets for the drugs are shown here. In total, 13 drug combinations have been predicted to be the top 10 potential combinations. Seven combinations have been confirmed to be synergistic by antifungal biological experiments.

Group	Drug 1	Target	Drug 2	Target	Synergy
1	Ketoconazole	ERG11, ergosterol biosynthesis	FK506	FKBP12	Y
2	Fluconazole	ERG11, ergosterol biosynthesis	Amphotericin B	Binding to Ergosterol to disrupt the cell membrane	N
3	Ketoconazole	ERG11, ergosterol biosynthesis	Terbinafine	ERG1, ergosterol biosynthesis	N
4	Ketoconazole	ERG11, ergosterol biosynthesis	Flucytosine	DNA or RNA	N
5	Fluconazole	ERG11, ergosterol biosynthesis	Radicicol	Hsp90	Y
6	Fluconazole	ERG11, ergosterol biosynthesis	Disulfiram	ABC transporters	N
7	Fluconazole	ERG11, ergosterol biosynthesis	Beauvericin	ABC transporters	Y
8	Voriconazole	ERG11, ergosterol biosynthesis	Caspofungin	1,3-β glucan synthase	Y
9	Ketoconazole	ERG11, ergosterol biosynthesis	Caspofungin	1,3-β glucan synthase	N
10	Posaconazole	ERG11, ergosterol biosynthesis	Terbinafine	ERG1, ergosterol biosynthesis	Y
11	Ketoconazole	ERG11, ergosterol biosynthesis	Micafungin	1,3-β glucan synthase	N
12	Itraconazole	ERG11, ergosterol biosynthesis	Lovastatin	HMG-Co-A reductase	Y
13	Fluconazole	ERG11, ergosterol biosynthesis	Geldanamycin	Hsp90	Y

### Synergistic drug combinations

Among the predictions, 7 pairs showed synergistic effects. For the Group 1 in Fig 6, FK506 is a novel immunosuppressant isolated from Streptomyces [45] and has been demonstrated to bind to FKBP12 to inhibit calcineurin [46], which is the key pathway for the cells responding to different stresses [47–50]. Radicicol and geldeanamycin from Groups 5 and 13, respectively, can bind to Hsp90 and alter its function. Also, Hsp90 can act as the molecular chaperone to calcineurin [51,52]. Synergistic activity between FK506 and ketoconazole occurred at 16 h (Fig 6 and S14 Table), but only at 48 h for radicicol with fluconazole and geldeanamycin with fluconazole (Fig 6, S15 and S16 Tables).

Synergism between beauvericin and ketoconazole from Group 7 was identified based on our high-throughput synergistic screening platform [16]. Beauvericin can inhibit drug efflux pumps to reduce the accumulation of azoles and present synergistic activity [53], and the synergistic activity was observed at 48 h (Fig 6 and S17 Table).

In Group 8, caspofungin is a lipopeptide antifungal drug that inhibits the enzyme β(1,3)-D-Glucan synthase, thus interfering with the integrity of the fungal cell wall [54]. Our results proved that it could synergize with voriconazole and that synergistic activity started at 24 h (Fig 6 and S18 Table).

In Group 10, posaconazole is a triazole antifungal drug that inhibits lanosterol 14α-demethylase (ERG11) to block ergosterol biosynthesis [55,56]. Terbinifine also inhibits ergosterol biosynthesis by inhibiting squa lene epoxidase (ERG1) [57]. These two drugs, which have the same pathway, showed synergistic activity that occurred at 24 h (Fig 6 and S19 Table).

Lovastatin from Group 12 is a member of the drug class of statins, used for lowering cholesterol by inhibiting the 3-hydroxy-3methylglutaryl-coenzyme A reductase (HMG-CoA reductase), an enzyme that catalyzes the conversion of HMG-CoA to mevalonate [58]. Lovastatin could synergize with itraconazole in this study, and the synergistic activity started at 16 h (Fig 6 and S20 Table).

### Non-synergistic drug combinations

We found that 6 pairs of the predictions showed non-synergistic synergistic effects. Amphotericin B in Group 2 is a kind of polyene antifungal drug by binding to ergosterol to destroy the cell membrane [59,60]. Some researchers observed that a combination of azoles (such as posaconazole and itraconazole) can decrease the fungal infection burden [61–63]. However, others have observed no difference using a combination of ketoconazole or fluconazole and amphotericin B [64–66]. Ayse et al. reported that some isolates even showed antagonistic activities between amphotericin B and fluconazole [67]. Our results proved the absence of synergistic activity, even after 48 h (S5 Fig and S21 Table).

In Group 3, no synergistic activity was observed between terbinafine and ketoconnazole on *C*. *albicans*, which is in agreement with a previous report [68]. However, it does show synergism with itraconazole [16,69,70], fluconazole, voriconazole [71] and posaconazole (S5 Fig and S22 Table).

Flucytosine from Group 4 is a fluorinated pyrimidine analogue, and it inhibits fungal RNA and DNA synthesis [72]. The combination of ketoconazole and flucytosine can increase the antifungal effect, but no synergistic activity was observed [73]. Our result demonstrated that this combination only had “additive effect” (0.5<FICI<1), but no synergistic activity (S5 Fig and S23 Table).

Disulfiram in Group 6 is an antifungal and can inhibit the drug efflux pump from *C*. *albicans* [74]. Ann et al. found antagonistic activities in clinical isolates when combining disulfiram with fluconazole [75]. Our results show that the combination of fluconazole and disulfiram had no synergistic activity during two days of incubation (S5 Fig and S24 Table).

Micafungin, the second available agent in the echinocandins class, is a potent inhibitor of 1,3-β-D-glucan synthase. It is used against fungal pathogens, such as Candida spp. and Aspergillus spp [76]. Our result showed that the combination of voriconazole and caspofungin had synergistic activity (Group 8), but micafungin did not show synergy with ketoconazole (Group 11, S5 Fig and S25 Table) during two days of incubation, indicating that the interactions between echinocandins and triazoles may be related to the chemical structures of individual agents.

For the same reason, in this study, we confirmed that caspofungin has synergistic activity with voricanazole (Group 8). However, the combination of ketoconazole and caspofungin had no synergistic activity (Group 9, S5 Fig and S26 Table), just like the nonsynergistic combination of ketoconazole and micafungin (Group 11, S5 Fig and S25 Table), suggesting that the synergistic activity may be related to chemical structures.

## Discussion

Drug combinations represent a promising strategy for overcoming fungal drug resistance and treating complex diseases. In this work, NLLSS was developed to predict potential synergistic drug combinations by integrating known synergistic drug combinations, unlabeled drug combinations, drug-target interactions, and drug chemical structures on a large scale. NLLSS was motivated based on the observation that principal drugs which obtain synergistic effect with similar adjuvant drugs are often similar and vice versa. Both cross validations and experimental validations indicated that NLLSS has an excellent performance of identifying potential synergistic drug combinations. Out of 13 predicted antifungal synergistic drug combinations, 7 candidates were experimentally confirmed. NLLSS could provide a new strategy to identify potential synergistic antifungal combinations, explore new indications of existing drugs, and provide useful insights into the underlying molecular mechanisms of synergistic drug combinations.

Previous research about synergistic drug combinations could be divided into the following three categories: only give the definition of synergy to determine whether or not a given drug combination is synergistic, only implemented experiments to screen synergistic combinations, and only implemented computational predictions to provide potential synergistic combinations. For example, methods such as combination index equation, Loewe additive model, HAS model, and universal response surface approach, only defined the concept of synergy, measured dose-effect curves, and determined whether or not a given drug combination is synergistic. Further, plenty of previous research has been devoted to implementing drug combination screening to search synergistic combinations and certain previous methods [14,28,77] strongly rely on experimental results, our method predicts synergistic drug combinations based only on the information from databases and the scientific literatures. Finally, although some computational methods has been developed, such as the model developed by Li et al. [29], Zhao et al. [30], Yin et al. [33], Huang et al. [31], Huang et al. [32], Iwata et al [34], and Chen et al. [35], no experimental validation could be seen in their studies. Therefore, NLLSS differs significantly from previous methods based on the following four aspects, which also constitute the success factors of NLLSS. First, chemical structure information, drug-target interactions, and known synergistic drug combinations were integrated to capture potential synergistic associations. Second, known experimentally verified synergistic drug combinations were used as a seed dataset for predicting potential candidate combinations. Furthermore, a semi-supervised technique was adopted, whose advantage over supervised methods has been shown in many previous studies. More importantly, we not only developed computational models to quantitatively identify potential synergistic drug combination candidates, but also implemented experimental validations. These features highlight that NLLSS is essentially different from most of previous methods for drug combination predictions. Furthermore, the drug combination exploration space of NLLSS could include any drugs even if they do not have any known synergetic partners. Based on the procedure of NLLSS and drug combination similar nature proposed in this study, one drug could have predicted synergistic drug partners so long as it has at least one similar drug and this similar drug has been involved in known synergistic drug combinations. As mentioned, we implemented 10-fold, 5-fold, and 3-fold CV in all the three datasets, respectively. In this case, many drugs would do not have any known synergistic partners in the training samples. As the results listed in Table 2, NLLSS has a reliable and robust performance in all the validation schemas, which could indicated NLLSS could be effectively applied to the drugs without any known synergistic drug combination partners.

However, some limitations of NLLSS should be mentioned. First, the performance of NLLSS could be further improved by more available known synergistic drug combinations and drug-target interactions. Second, a more reliable measure of drug similarity would improve NLLSS. To do this, more biological information should be integrated to measure drug similarity. We also plan to develop new similarity integration methods to integrate different similarity measures for the further performance improvement. Furthermore, drugs must be classified into principal drug and adjuvant drug before predicting potential combinations in our method. Currently, there is still no acknowledged dividing standard for principal drugs and adjuvant drugs. Furthermore, when two drugs in the combination were classified as both principal drug and adjuvant drug, the same drug combination will obtain two different synergistic probability scores based on NLLSS. In this paper, we only chose the greater score as the final synergistic probability score of such drug combinations. In this study, NLLSS was developed based on the framework of LapRLS, which used different drug similarity matrix to construct different classifiers. Therefore, we can’t directly obtain a single classifier from the start. If we introduced the information of principal drug similarity matrix and adjuvant drug similarity matrix into the same cost function, we can’t obtain the analytical solution of the corresponding optimization problem. In the future, we would develop new computational tool, which could construct a single classifier in the beginning. Finally, some drug combinations are composed of more than 2 drugs. The current version of NLLSS only can predict drug combinations consisting of 2 drugs. In future work, we will develop new tools and methods to overcome the limitations of NLLSS.

Finally, predictions based on NLLSS benefits the understanding of the mechanisms underlying synergistic drug combinations. For example, we predicted that the inhibitors from the calcineurin pathway and ergosterol biosynthesis pathway are the most popular synergistic combinations (Groups 1, 5 and 13, Fig 6). Calcineurin is a Ca 2+ / calmodulin-dependent serine/threonine phosphatase, and its structure and activation pathways are highly conserved from yeast to high eukaryotes [78]. In *C*. *albicans*, some reports revealed the involvement of calcineurin in antifungal tolerance, cell morphogenesis and virulence. The deletion of genes from the calcineurin pathway resulted in loss of tolerance to several antifungal agents, such as fluconazole, terbinafine, inhibitors for ergosterol biosynthesis, and caspofungin, an inhibitor of cell wall biosynthesis, and other growth-inhibiting agents (e.g., fluphenazine, caffeine) [78]. When fungal cells are treated by triazole drugs, it is possible that cells incur membrane damage, as well as the accumulation of toxic sterols, and, at this time, the calcineurin pathway is activated to respond to these stresses [47–50,79]. The use of calcineurin pathway inhibitors makes fungal cells vulnerable to triazole drugs (Fig 6). These results indicate that many more synergistic antifungal combinations can be discovered from these two pathways.

It is not clear that how lovastatin affects *C*. *albicans*, but it was reported that fluvastatin, the analog of lovastatin, has synergistic effect with itraconazole [51,80,81]. We proved that lovastatin can also synergize with itraconazole (Group 12, Fig 6 and S20 Table) and that lovastatin may act in a similar manner with fluvastatin. It is interesting that two inhibitors (Group 10: posaconazole and terbinafine) from the ergosterol biosynthesis pathway have synergistic antifungal activity (Fig 6 and S19 Table). Terbinafine targets ERG1, which is the upstream gene for ERG11, the target for posaconazole. Based on our predictions, it is the only synergistic combination that targets the same pathway. However, ketoconazole does not show synergistic effect with terbinafine (Group 3, S5 Fig and S22 Table), indicating either that the synergistic activity results from the different chemical structure of triazoles or that triazoles have some other effects on fungal cells, such as mitochondria [82].

Synergistic antifungal activities from groups 1 and 12 can be observed at 16 h, and they maintain their activity, even after 48 h, but group 8 showed synergistic activity after 24 h incubation. The synergistic activities of groups 5, 7 and 13 can only be observed at 48h. The synergistic activity from group 8 only can be observed at 24 h (Fig 6). These time-course studies provide important information for the application of these synergistic combinations.

## Supporting Information

S1 Fig Schematic illustrating the similar nature of synergistic drug combinations. Blue nodes represent principal drugs, and yellow nodes represent adjuvant drugs. The arc from the principal drug to the adjuvant drug means that two drugs have synergistic effect when combined in the antifungal assays. The edge between two principal (adjuvant) drugs represents the similarity between two drugs. Thickness of edges linking drugs indicates degree of similarity between them. This figure shows that principal drugs which obtain synergistic effect with similar adjuvant drugs are often similar. Similar adjuvant drugs, Amphotericin and Retigeric acid B (RAB), obtain synergistic effect with eight principal drugs, including Caspofungin, Disulfiram, Flucytosine, Itraconazole, Ketoconazole, Micafungin, Terbinafine, and Fluconazole. It can be observed that those eight principal drugs are similar. Also the principal drugs which obtain synergistic effect with Tamoxifen are similar and form a module. On the contrary, most of the principal drugs which obtain the synergistic effect with dissimilar adjuvant drugs (Amphotericin and Tamoxifen, Retigeric acid B and Tamoxifen) are dissimilar.
(TIF)

S2 Fig In order to calculate the similarity between two drugs, we extract the information from known drug synergistic combinations. The underlying assumption is that if two principal (adjuvant) drugs obtain synergistic effect with more common adjuvant (principal) drugs, they have greater similarity.
(TIF)

S3 Fig LOOCV was implemented in the antifungal synergistic drug combinations in dataset 2. AUC was calculated to evaluate the performance of the method. Here known synergistic drug combinations were used as the test dataset. The ROC curves of NLLSS based on the combination of two classifiers and based only on a single classifier were compared. The results confirmed the performance advantage of combining the classifiers in the principal and adjuvant drug space into a single classifier.
(TIF)

S4 Fig LOOCV was implemented in the antifungal synergistic drug combinations in dataset 3. AUC was calculated to evaluate the performance of the method. Here known synergistic drug combinations were used as the test dataset. The ROC curves of NLLSS based on the combination of two classifiers and based only on a single classifier were compared. The results confirmed the performance advantage of combining the classifiers in the principal and adjuvant drug space into a single classifier.
(TIF)

S5 Fig Nonsynergistic antifungal combinations: The x and y axis indicated the concentrations of the combination drugs used in the synergistic screening. The dots were the active concentrations for inhibiting the growth of C. albicans in the combinations. All experiments were performed on 96-well plates and incubated at 35 oC for 48 h. The data from three independent experiments were measured at different time points (16 h, 24 h and 48 h).
(JPG)

S1 Table All drugs or compounds used in antifungal drug combination experiments are listed with their antifungal activity and related references. (XLS)

S2 Table All synergistic antifungal drug combinations from the literature are listed with related references. (XLS)

S3 Table All compounds or drugs used in the antifungal drug combination experiments against *Candida albicans* are listed with their antifungal activity and related references. (XLS)

S4 Table All the synergistic antifungal drug combinations against *Candida albicans* from the literature are listed with related references. (XLS)

S5 Table All compounds or drugs used in the antifungal azole drug combination experiments in all fungi are listed with their antifungal activity and related references. (XLS)

S6 Table All the synergistic antifungal azole drug combinations in all fungi from the literature are listed with related references. (XLS)

S7 Table Target proteins of all drugs in drug combination dataset 1. (XLS)

S8 Table Target proteins of all drugs in drug combination dataset 2. (XLS)

S9 Table Target proteins of all drugs in drug combination dataset 3. (XLS)

S10 Table Complete predictive results for potential antifungal synergistic drug combination in dataset 1 are listed. (XLS)

S11 Table Complete predictive results for potential antifungal synergistic drug combination in dataset 2 are listed. (XLS)

S12 Table Complete predictive results for potential antifungal synergistic drug combination in dataset 3 are listed. (XLS)

S13 Table The MICs of all the test drugs when used alone. (DOCX)

S14 Table MICs data for the synergistic antifungal combinations and their FIC index at different time points. The data were the active concentrations for inhibiting the growth of C. albicans in the combinations. All experiments were performed on 96-well plates and incubated at 35 oC for 48 h. Data from three independent experiments were measured at different time points (16 h, 24 h and 48 h). The FIC index was calculated by using the active concentrations in the two drug combinations compared with the active concentrations of each drug when used alone (these data for each group were listed in S13 Table).
(DOC)

S15 Table MICs data for the synergistic antifungal combinations and their FIC index at different time points. (DOC)

S16 Table MICs data for the synergistic antifungal combinations and their FIC index at different time points. (DOC)

S17 Table MICs data for the synergistic antifungal combinations and their FIC index at different time points. (DOC)

S18 Table MICs data for the synergistic antifungal combinations and their FIC index at different time points. (DOC)

S19 Table MICs data for the synergistic antifungal combinations and their FIC index at different time points. (DOC)

S20 Table MICs data for the synergistic antifungal combinations and their FIC index at different time points. (DOC)

S21 Table MICs data for the synergistic antifungal combinations and their FIC index at different time points. (DOC)

S22 Table MICs data for the synergistic antifungal combinations and their FIC index at different time points. (DOC)

S23 Table MICs data for the synergistic antifungal combinations and their FIC index at different time points. (DOC)

S24 Table MICs data for the synergistic antifungal combinations and their FIC index at different time points. (DOC)

S25 Table MICs data for the synergistic antifungal combinations and their FIC index at different time points. (DOC)

S26 Table MICs data for the synergistic antifungal combinations and their FIC index at different time points. (DOC)
