# Supplementary figures and images for: NLLSS: Predicting Synergistic Drug Combinations Based on Semi-supervised Learning
Source: PLoS Comput Biol. 2016 Jul 14;12(7):e1004975. doi: 10.1371/journal.pcbi.1004975 (PMC4945015; doi:10.1371/journal.pcbi.1004975)

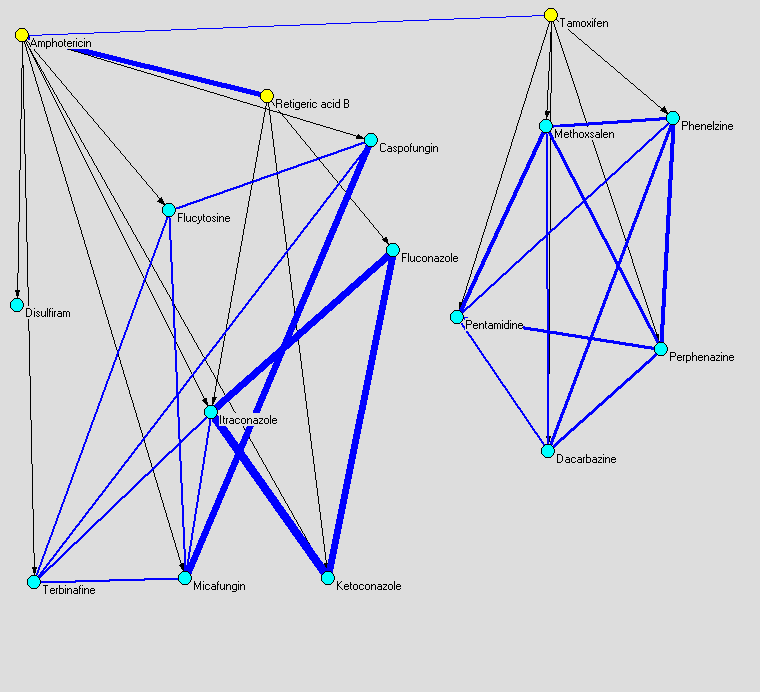

Supplement: S1 Fig — Blue nodes represent principal drugs, and yellow nodes represent adjuvant drugs. The arc from the principal drug to the adjuvant drug means that two drugs have synergistic effect when combined in the antifungal assays. The edge between two principal (adjuvant) drugs represents the similarity between two drugs. Thickness of edges linking drugs indicates degree of similarity between them. This figure shows that principal drugs which obtain synergistic effect with similar adjuvant drugs are often similar. Similar adjuvant drugs, Amphotericin and Retigeric acid B (RAB), obtain synergistic effect with eight principal drugs, including Caspofungin, Disulfiram, Flucytosine, Itraconazole, Ketoconazole, Micafungin, Terbinafine, and Fluconazole. It can be observed that those eight principal drugs are similar. Also the principal drugs which obtain synergistic effect with Tamoxifen are similar and form a module. On the contrary, most of the principal drugs which obtain the synergistic effect with dissimilar adjuvant drugs (Amphotericin and Tamoxifen, Retigeric acid B and Tamoxifen) are dissimilar. (TIF) [file pcbi.1004975.s001.tif]

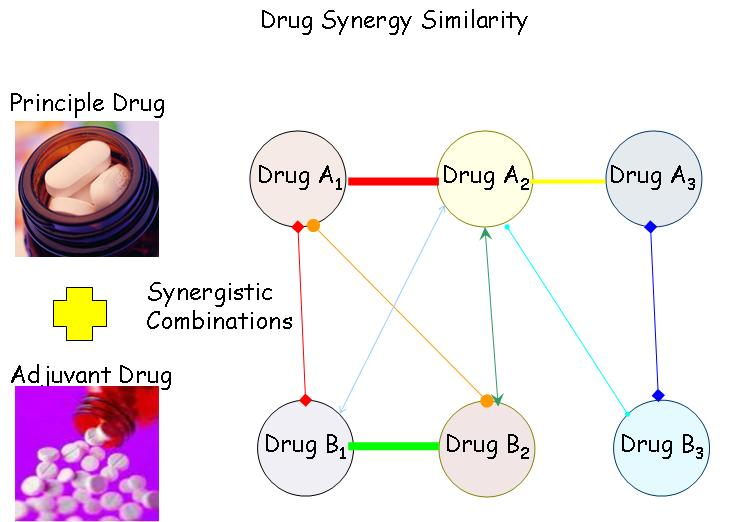

Supplement: S2 Fig — The underlying assumption is that if two principal (adjuvant) drugs obtain synergistic effect with more common adjuvant (principal) drugs, they have greater similarity. (TIF) [file pcbi.1004975.s002.tif]

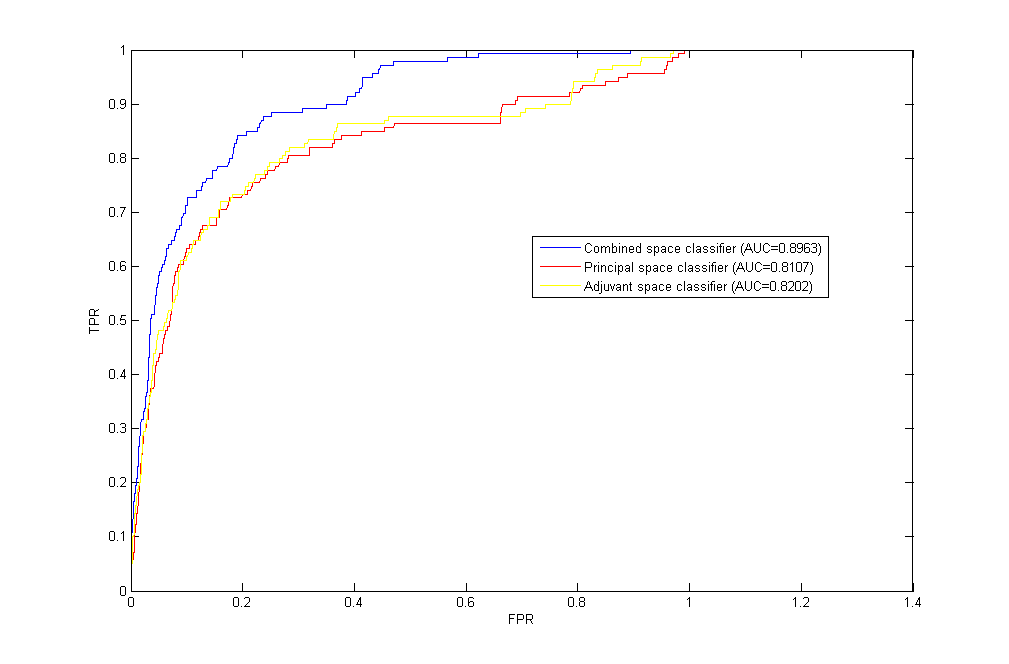

Supplement: S3 Fig — AUC was calculated to evaluate the performance of the method. Here known synergistic drug combinations were used as the test dataset. The ROC curves of NLLSS based on the combination of two classifiers and based only on a single classifier were compared. The results confirmed the performance advantage of combining the classifiers in the principal and adjuvant drug space into a single classifier. (TIF) [file pcbi.1004975.s003.tif]

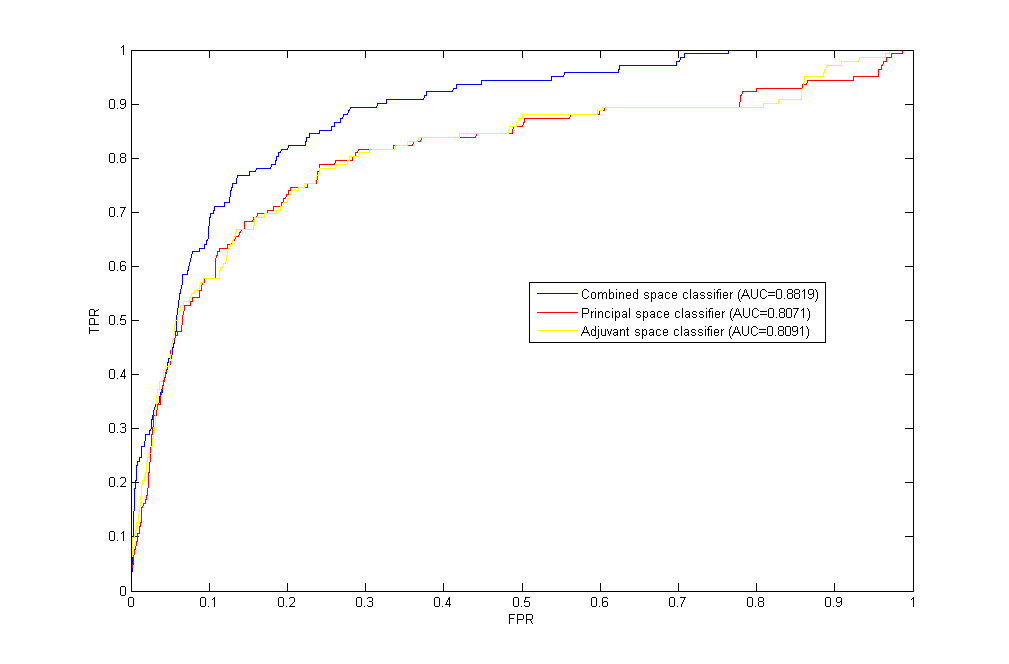

Supplement: S4 Fig — AUC was calculated to evaluate the performance of the method. Here known synergistic drug combinations were used as the test dataset. The ROC curves of NLLSS based on the combination of two classifiers and based only on a single classifier were compared. The results confirmed the performance advantage of combining the classifiers in the principal and adjuvant drug space into a single classifier. (TIF) [file pcbi.1004975.s004.tif]

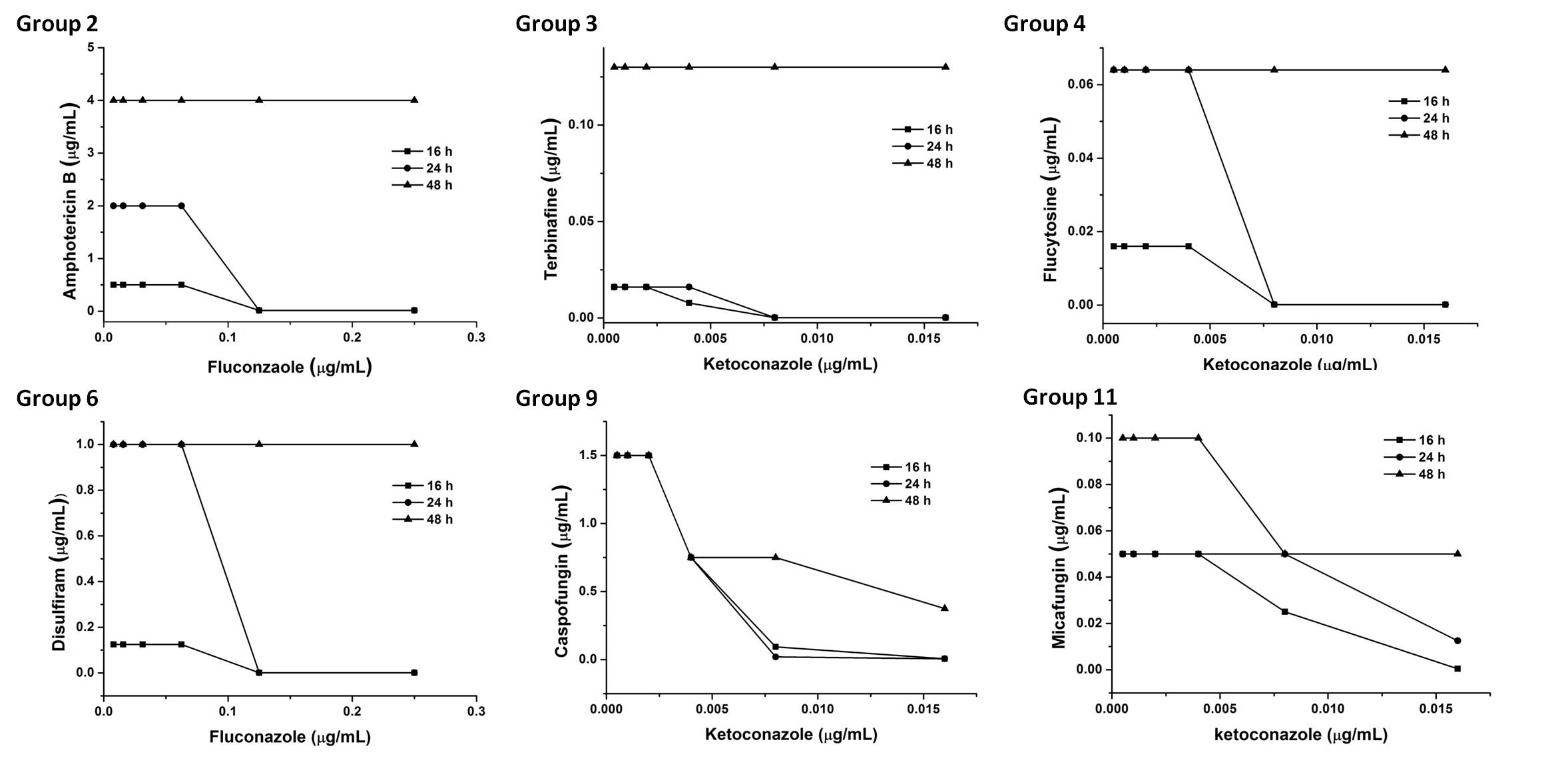

Supplement: S5 Fig — The dots were the active concentrations for inhibiting the growth of C. albicans in the combinations. All experiments were performed on 96-well plates and incubated at 35 oC for 48 h. The data from three independent experiments were measured at different time points (16 h, 24 h and 48 h). (JPG) [file pcbi.1004975.s005.jpg]
