# Supplementary material for: NLLSS: Predicting Synergistic Drug Combinations Based on Semi-supervised Learning
Source: PLoS Comput Biol. 2016 Jul 14;12(7):e1004975. doi: 10.1371/journal.pcbi.1004975 (PMC4945015; doi:10.1371/journal.pcbi.1004975)
Supplement: S13 Table — (DOCX) [file pcbi.1004975.s018.docx]

| Drugs | MIC （μg/mL） | | |
| --- | --- | --- | --- |
|  | 16h | 24h | 48h |
| Ketoconazole | 0.008~0.016 | 0.016 | >4 |
| Fluconazole | 0.125~0.25 | 0.25 | 100 |
| Voriconazole | 0.004 | 0.008-0.016 | >4 |
| Posaconazole | 0.004-0.008 | 0.008 | >8 |
| Itraconazole | 0.016~0.032 | 8-16 | >16 |
| Amphotericin B | 1 | 2 | 4 |
| Beauvericin | 4 | 4 | 8 |
| FK506 | >100 | >100 | >100 |
| Terbinafine | 0.016 | 0.03125-0.0625 | >0.125 |
| Flucytosine | 0.016 | 0.064 | >0.064 |
| Radicicol | 6.25 | 6.25 | 12.5 |
| Disulfiram | 0.125~0.25 | 1 | >1 |
| Lovastatin | >50 | >50 | >50 |
| Geldanamycin | 12.5 | 25 | 25 |
| Caspofungin | 1.5 | 1.5 | 1.5 |
| Micafungin | 0.05 | 0.05 | 0.05 |
