# Supplementary material for: NLLSS: Predicting Synergistic Drug Combinations Based on Semi-supervised Learning
Source: PLoS Comput Biol. 2016 Jul 14;12(7):e1004975. doi: 10.1371/journal.pcbi.1004975 (PMC4945015; doi:10.1371/journal.pcbi.1004975)
Supplement: S14 Table — The data were the active concentrations for inhibiting the growth of C. albicans in the combinations. All experiments were performed on 96-well plates and incubated at 35 oC for 48 h. Data from three independent experiments were measured at different time points (16 h, 24 h and 48 h). The FIC index was calculated by using the active concentrations in the two drug combinations compared with the active concentrations of each drug when used alone (these data for each group were listed in S13 Table). (DOC) [file pcbi.1004975.s019.doc]

| Ketoconazole | 16h | | 24h | | 48h | |
| --- | --- | --- | --- | --- | --- | --- |
|  | FK506 | FIC Index | FK506 | FIC Index | FK506 | FIC Index |
| 0.032 | <0.098 | >2 | <0.098 | >2 | <0.098 | <1 |
| 0.016 | <0.098 | >1 | <0.098 | >1 | 0.20 | <0.5 |
| 0.008 | <0.098 | 0.5-1 | <0.098 | 0.5-1 | 0.39 | <0.26 |
| 0.004 | 0.39 | <0.5 | 0.39 | <0.5 | 0.78 | <0.14 |
| 0.002 | 50 | <0.5 | >50 | >1 | >50 | >1 |
| 0.001 | 50 | <0.5 | >50 | >1 | >50 | >1 |
| 0.0005 | 50 | <0.5 | >50 | >1 | >50 | >1 |
