# Supplementary material for: NLLSS: Predicting Synergistic Drug Combinations Based on Semi-supervised Learning
Source: PLoS Comput Biol. 2016 Jul 14;12(7):e1004975. doi: 10.1371/journal.pcbi.1004975 (PMC4945015; doi:10.1371/journal.pcbi.1004975)
Supplement: S16 Table — (DOC) [file pcbi.1004975.s021.doc]

| Fluconazole | 16h | | 24h | | 48h | |
| --- | --- | --- | --- | --- | --- | --- |
|  | GDM* | FIC Index | GDM | FIC Index | GDM | FIC Index |
| 0.5 | <0.049 | >2 | <0.049 | >2 | 0.098 | <1 |
| 0.25 | <0.049 | >1 | <0.049 | >1 | 0.39 | <0.5 |
| 0.13 | <0.049 | 0.5-1 | <0.049 | 0.5-1 | 6.25 | <0.5 |
| 0.063 | 12.5 | >1 | 25 | >1 | >25 | >1 |
| 0.031 | 12.5 | >1 | >25 | >1 | >25 | >1 |
| 0.016 | 12.5 | >1 | >25 | >1 | >25 | >1 |
| 0.0078 | 12.5 | >1 | >25 | >1 | >25 | >1 |

_*_: GDM: geldanamycin
