# Supplementary material for: NLLSS: Predicting Synergistic Drug Combinations Based on Semi-supervised Learning
Source: PLoS Comput Biol. 2016 Jul 14;12(7):e1004975. doi: 10.1371/journal.pcbi.1004975 (PMC4945015; doi:10.1371/journal.pcbi.1004975)
Supplement: S17 Table — (DOC) [file pcbi.1004975.s022.doc]

| Fluconazole | 16h | | 24h | | 48h | |
| --- | --- | --- | --- | --- | --- | --- |
|  | BEA* | FIC Index | BEA | FIC Index | BEA | FIC Index |
| 0.5 | <0.031 | >2 | <0.031 | >2 | 2 | <0.75 |
| 0.25 | <0.031 | >1 | <0.031 | >1 | 2 | ≤0.5 |
| 0.13 | <0.031 | 0.5-1 | <0.031 | 0.5-1 | 2 | <0.5 |
| 0.063 | 2-4 | 0.5-1 | 2-4 | 0.5-1 | 8 | >1 |
| 0.031 | 4 | >1 | 4 | >1 | 8 | >1 |
| 0.016 | 4 | >1 | 4 | >1 | 8 | >1 |
| 0.0078 | 4 | >1 | 4 | >1 | 8 | >1 |

_*_: BEA: beauvericin
