# Supplementary material for: NLLSS: Predicting Synergistic Drug Combinations Based on Semi-supervised Learning
Source: PLoS Comput Biol. 2016 Jul 14;12(7):e1004975. doi: 10.1371/journal.pcbi.1004975 (PMC4945015; doi:10.1371/journal.pcbi.1004975)
Supplement: S18 Table — (DOC) [file pcbi.1004975.s023.doc]

| Voriconazole | 16h | | 24h | | 48h | |
| --- | --- | --- | --- | --- | --- | --- |
|  | Caspo* | FIC Index | Caspo | FIC Index | Caspo | FIC Index |
| 0.016 | <0.0059 | >2 | <0.0059 | >1 | 0.19-0.38 | <0.5 |
| 0.008 | <0.0059 | >2 | <0.0059 | 0.5-1 | 0.38 | <0.5 |
| 0.004 | <0.0059 | >1 | 0.094-0.19 | <0.5 | 0.38-0.75 | 0.5-1 |
| 0.002 | 0.19-0.38 | 0.5-1 | 0.38-0.75 | 0.5-1 | 0.75 | 0.5-1 |
| 0.001 | 1.5 | >1 | 1.5 | >1 | 1.5 | >1 |
| 0.0005 | 1.5 | >1 | 1.5 | >1 | 1.5 | >1 |
| 0.00025 | 1.5 | >1 | 1.5 | >1 | 1.5 | >1 |

_*_: Caspo: caspofungin
