# Supplementary material for: NLLSS: Predicting Synergistic Drug Combinations Based on Semi-supervised Learning
Source: PLoS Comput Biol. 2016 Jul 14;12(7):e1004975. doi: 10.1371/journal.pcbi.1004975 (PMC4945015; doi:10.1371/journal.pcbi.1004975)
Supplement: S20 Table — (DOC) [file pcbi.1004975.s025.doc]

| Itraconazole | 16h | | 24h | | 48h | |
| --- | --- | --- | --- | --- | --- | --- |
|  | Lovas* | FIC Index | Lovas | FIC Index | Lovas | FIC Index |
| 0.032 | <0.098 | >1 | <0.098 | <1 | 6.25 | <1 |
| 0.016 | <0.098 | 0.5-1 | 1.6 | <0.5 | 6.25 | <0.5 |
| 0.008 | 1.6 | <0.28 | 6.25 | <0.28 | 6.25 | <0.38 |
| 0.004 | 6.25 | <0.25 | 12.5 | <0.25 | 12.5 | <0.38 |
| 0.002 | 12.5-25 | 0.5-1 | 25 | 0.5-1 | 25 | 0.5-1 |
| 0.001 | 25-50 | 0.5-1 | 50 | 0.5-1 | >50 | 0.5-1 |
| 0.0005 | >50 | >1 | >50 | 0.5-1 | >50 | 0.5-1 |

_*_: Lovas: lovastatin
