# Supplementary material for: NLLSS: Predicting Synergistic Drug Combinations Based on Semi-supervised Learning
Source: PLoS Comput Biol. 2016 Jul 14;12(7):e1004975. doi: 10.1371/journal.pcbi.1004975 (PMC4945015; doi:10.1371/journal.pcbi.1004975)
Supplement: S23 Table — (DOC) [file pcbi.1004975.s028.doc]

| Ketoconazole | 16h | | 24h | | 48h | |
| --- | --- | --- | --- | --- | --- | --- |
|  | Flucy* | FIC Index | Flucy | FIC Index | Flucy | FIC Index |
| 0.032 | <0.00013 | >2 | <0.00013 | >2 | >0.064 | >2 |
| 0.016 | <0.00013 | >1 | <0.00013 | >1 | >0.064 | >2 |
| 0.008 | <0.00013 | 0.5-1 | <0.00013 | 0.5-1 | >0.064 | >2 |
| 0.004 | 0.016 | >1 | 0.064 | >1 | >0.064 | >2 |
| 0.002 | 0.016 | >1 | 0.064 | >1 | >0.064 | >2 |
| 0.001 | 0.016 | >1 | 0.064 | >1 | >0.064 | >2 |
| 0.0005 | 0.016 | >1 | 0.064 | >1 | >0.064 | >2 |

_*_: Flucy: flucytosine
