# Supplementary material for: NLLSS: Predicting Synergistic Drug Combinations Based on Semi-supervised Learning
Source: PLoS Comput Biol. 2016 Jul 14;12(7):e1004975. doi: 10.1371/journal.pcbi.1004975 (PMC4945015; doi:10.1371/journal.pcbi.1004975)
Supplement: S25 Table — (DOC) [file pcbi.1004975.s030.doc]

| Ketoconazole | 16h | | 24h | | 48h | |
| --- | --- | --- | --- | --- | --- | --- |
|  | Mica* | FIC Index | Mica | FIC Index | Mica | FIC Index |
| 0.032 | <0.00039 | >2 | <0.00039 | >2 | 0.05 | >1 |
| 0.016 | <0.00039 | >1 | 0.013-0.025 | >1 | 0.05 | >1 |
| 0.008 | 0.025-0.05 | >1 | 0.05 | >1 | 0.05 | >1 |
| 0.004 | 0.05 | >1 | 0.05 | >1 | 0.1 | >2 |
| 0.002 | 0.05 | >1 | 0.05 | >1 | 0.1 | >2 |
| 0.001 | 0.05 | >1 | 0.05 | >1 | 0.1 | >2 |
| 0.0005 | 0.05 | >1 | 0.05 | >1 | 0.1 | >2 |

_*_: Mica: micafungin
